# Emerging Role of Translational Digital Biomarkers Within Home Cage Monitoring Technologies in Preclinical Drug Discovery and Development

**DOI:** 10.3389/fnbeh.2021.758274

**Published:** 2022-02-14

**Authors:** Szczepan W. Baran, Natalie Bratcher, John Dennis, Stefano Gaburro, Eleanor M. Karlsson, Sean Maguire, Paul Makidon, Lucas P. J. J. Noldus, Yohann Potier, Giorgio Rosati, Matt Ruiter, Laura Schaevitz, Patrick Sweeney, Megan R. LaFollette

**Affiliations:** ^1^Novartis Institutes for BioMedical Research, Cambridge, MA, United States; ^2^Office of Global Animal Welfare, AbbVie, North Chicago, IL, United States; ^3^United States Food and Drug Administration, Silver Spring, MD, United States; ^4^Tecniplast S.p.A., Buguggiate, Italy; ^5^Calico Life Sciences LLC, South San Francisco, CA, United States; ^6^GlaxoSmithKline, Collegeville, PA, United States; ^7^Comparative Medicine, AbbVie, South San Francisco, CA, United States; ^8^Noldus Information Technology BV, Wageningen, Netherlands; ^9^Department of Biophysics, Radboud University, Nijmegen, Netherlands; ^10^Tessera Therapeutics Inc., Cambridge, MA, United States; ^11^Unified Information Devices Inc., Lake Villa, IL, United States; ^12^Recursion Pharmaceuticals Inc., Salt Lake City, UT, United States; ^13^Actual Analytics Ltd., Edinburgh, United Kingdom; ^14^Naason Science, Inc., Cheongju-si, South Korea; ^15^The North American 3Rs Collaborative, Denver, CO, United States

**Keywords:** 3Rs (reduce replace refine), digital biomarkers, translation, preclinical, drug discovery and development, home cage, rodents

## Abstract

In drug discovery and development, traditional assessment of human patients and preclinical subjects occurs at limited time points in potentially stressful surroundings (i.e., the clinic or a test arena), which can impact data quality and welfare. However, recent advances in remote digital monitoring technologies enable the assessment of human patients and preclinical subjects across multiple time points in familiar surroundings. The ability to monitor a patient throughout disease progression provides an opportunity for more relevant and efficient diagnosis as well as improved assessment of drug efficacy and safety. In preclinical *in vivo* animal models, these digital technologies allow for continuous, longitudinal, and non-invasive monitoring in the home environment. This manuscript provides an overview of digital monitoring technologies for use in preclinical studies including their history and evolution, current engagement through use cases, and impact of digital biomarkers (DBs) on drug discovery and the 3Rs. We also discuss barriers to implementation and strategies to overcome them. Finally, we address data consistency and technology standards from the perspective of technology providers, end-users, and subject matter experts. Overall, this review establishes an improved understanding of the value and implementation of digital biomarker (DB) technologies in preclinical research.

## Introduction

Drug discovery and development is under tremendous pressure to accelerate the production and delivery of novel, safe, and effective therapies to patients, which depends on collaboration among the pharmaceutical industry, academic collaborators, contract research organizations, technology providers, and regulatory agencies. Thus, the field is reimagining drug discovery and development by leveraging digital transformation with emerging technologies. Initial efforts in preclinical research involved automating animal behavior observations with analog devices in the 1980s (Donát, 1991), followed by extracting metrics from videos in the early 1990s (Spruijt et al., 1992; Sams-Dodd, 1995; Noldus et al., 2001). Automated systems have since been commonly used to quantify rodent behavior in a wide variety of test paradigms (Crawley, 2007; Carter and Shieh, 2015). These traditional behavioral measures are collected by removing animals from their home cage and placing them in temporary enclosures, which, along with even routine husbandry, may affect behavioral and physiological parameters (Saibaba et al., 1996; Balcombe et al., 2004; Schreuder et al., 2007; Meller et al., 2011; Gerdin et al., 2012). Removal from the home cage can also cause stress, negatively impacting animal welfare and scientific data quality.

To mitigate these negative effects, technologies are being developed to collect digital biomarkers (DBs) from animals while in their home cage environment. Thus far, results from these technologies confirm earlier findings that handling and removing animals from home cages change their behavior and physiology (Lim et al., 2019; Pernold et al., 2019; Baran et al., 2020). For the purpose of this manuscript, home cage and home environment are defined as cages and environment where the animals are housed for the majority of their lifetime while in the vivarium (see Box 1), whereas DB refers to data collected continuously from unrestrained and un-instrumented animals in the home cage environment. In this context, un-instrumented may include animals with radio frequency identification (RFID) chips injected subcutaneously but not devices implanted surgically [National Research Council (US) Committee for the Update of the Guide for the Care and Use of Laboratory Animals, 2011]. Furthermore, this manuscript focuses on scalable (ability to monitor hundreds to thousands of animals in their home environment) and commercially available technologies, with the exception of historical perspective where other technologies are included.

**Box 1. Helpful definitions related to digital biomarkers.**

**Digital biomarker:** data collected continuously from unrestrained and un-instrumented animals in their home cage environment. These animals should not have undergone minor or major surgery with the exception of radio frequency identification (RFID) chips injected subcutaneously [National Research Council (US) Committee for the Update of the Guide for the Care and Use of Laboratory Animals, 2011].

**Translational digital biomarker (TDB)**: an objective, quantifiable measure of physiological and/or behavioral response to disease progression or therapeutic intervention that is collected by means of digital monitoring technologies, including both internal (e.g., injectable or ingestible) and external (e.g., wearable, camera, or electromagnetic field detector) sensors, which is clinically relevant and translate between preclinical studies and the clinic.

**Home cage or home environment:** cages and environment where animals are housed for the majority of their lifetime in the vivarium.

**Bench top cage or technology:** cages and technology (experimental test environments) not designed for permanent housing but where animals are housed for a short (from hours up to few days) period of time.

**Scalable:** ability to monitor hundreds to thousands of animals within a home environment.

**Technology verification:** ensuring, through demonstration of precision, reliability and reproducibility, that a device is measuring and storing data accurately.

**Analytical validation:** entails evaluation of data processing algorithms that convert technology-collected measurements into outputted metrics (Goldsack et al., 2020).

**Clinical validation:** accomplished by demonstrating that technology adequately identifies, measures, or predicts a meaningful clinical, biological, physical, functional state or experience in the specified (1) animal cohort and (2) context of use (Goldsack et al., 2020).

Currently, the technology and use of DBs in a home cage is emerging and is still in early stages of development and implementation, with recent advances allowing for longitudinal and scalable digital monitoring of rodents across a range of disease models including neural, psychiatric, respiratory, and oncology (Defensor et al., 2019; Baran et al., 2020, 2021; Do et al., 2020; Golini et al., 2020; Hobson et al., 2020; Shenk et al., 2020; Voikar and Gaburro, 2020; Grieco et al., 2021). These emerging technologies provide an opportunity to modernize animal assessment and refine how preclinical *in vivo* data are collected, analyzed, and visualized. Furthermore, these technologies have promising applications in efficacy and safety studies by improving translation and accelerating the delivery of better drug candidates into the clinic.

However, several challenges remain before these technologies are routinely implemented in drug discovery and development. Overcoming the challenges of onboarding and establishing robust qualification packages built around specific contexts of use (COU) is one of the highest priorities. To address these challenges, a group of stakeholders came together under the North American 3Rs Collaborative (NA3RsC) to establish the Translational Digital Biomarkers Initiative^1^. This Initiative is collaboration among pharmaceutical and biotechnology companies, technology providers, and other subject matter experts to improve understanding of the value and best practices for the implementation of scalable DB technologies in research. Its goal is to increase the adoption of translational DBs to advance the 3Rs.

The Translational Digital Biomarkers Initiative collectively defines a translational digital biomarker (TDB) as an objective, quantifiable measure of physiological and behavioral response to disease progression or therapeutic intervention that is collected by means of digital monitoring technologies, including both internal (e.g., injectable or ingestible) or external sensors (e.g., wearable, camera, or electromagnetic field detector), which are clinically relevant and translate between preclinical studies and the clinic. The TDB Initiative recognizes that these technologies are in the early stage of development and still require validation, characterization, qualification, and further evolution.

This manuscript provides an overview of TDB, including current challenges, gaps, and onboarding strategies. We aim to share end-user perspectives on evaluating and characterizing digital biomarkers (DBs) to support drug discovery and development, including describing likely COU where the pharmaceutical industry will incorporate DBs. We offer further insight into how these technologies will be applied in drug discovery and development for long-term impact on science and 3Rs. The intent of sharing this information is to expedite the engagement and integration of DBs into drug discovery and development.

## Evolution of Digital Biomarkers, From Short-Lasting Tests to Longitudinal Assessment in the Home Cage

### Behavior

**FIGURE 1:**
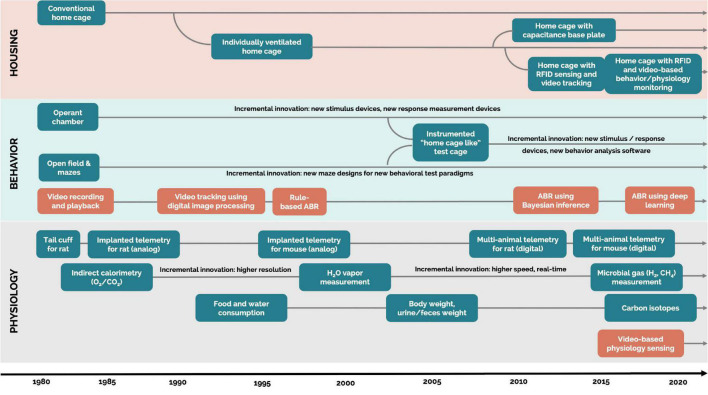
Evolution of technologies generating digital biomarkers of rodent behavior and physiology. Each arrow extending over 2020 is a technology that is currently available. Blue rectangles: hardware. Orange rectangles: software. ABR, automatic behavior recognition. Housing: systems that are designed for permanent housing of rodents in the vivarium. Home cage: cages where the animals are housed majority of their lifetime in the vivarium. Bench top cage or technology: cages and technology (experimental test environments) not designed for permanent housing but where the animals are housed for a short (from hours up to few days) period of time.

The evolution of automated measurement of rodent behavior began in the 1980s, progressing along with new technology (Figure 1). The first technologies to be used were photobeam activity monitors and video trackers (Donát, 1991). Then, as digital image processing and software-based video technologies were introduced, they significantly increased the implementation of DBs with bench top technologies (Spruijt et al., 1992). These systems allowed for more accurate and complex measurements, such as indicating time spent in specific zones of interest, distance traveled, velocity, acceleration, and social proximity (Sams-Dodd, 1995; Noldus et al., 2001; Spink et al., 2001).

In the present day, technology has progressed far beyond these simple measures in a single plane. For example, video tracking can even use machine learning to automatically classify specific postures and behaviors, such as grooming, rearing, sniffing, and walking, with the reliability of over 70% (on par with human observers) (Jhuang et al., 2010; van Dam et al., 2013). Deep learning for image processing also opens up exciting new avenues for innovation in automated behavioral observation (Mathis et al., 2018; Pereira et al., 2020), which is accelerated by animal-related open-source deep learning frameworks, libraries, and data sets (Serre, 2019; Mathis and Mathis, 2020; Mathis et al., 2020). With automated measurements of rodent behavior continuously improving, researchers have access to reliable and validated video tracking systems that generate a wealth of digital behavioral biomarkers. However, the majority of these systems require animals to be removed from their home cage environment and transferred into a test cage environment.

### Physiology

The evolution of automated measurement of rodent physiology also began in the early 1980s. Until then, blood pressure in rodents was measured with a cuff system (similar to human blood pressure measurement) applied to the tail in an anesthetized or restrained animal. However, in the 1990s, it was shown that restraint alone can increase blood pressure, thereby acting as a confounding variable (Bazil et al., 1993). To avoid this confounding variable, the majority of rodent cardiovascular studies are now performed on unrestrained, implanted telemetrized animals, as recommended by the American Cardiology Society (Anderson et al., 1999). These findings and recommendations related to blood pressure led to the development of additional technologies collecting physiological data, such as electrocardiogram (ECG), electroencephalography (EEG) and electromyography (EMG), blood pressure (BP), and blood glucose (BG). The majority of these technologies enable data collection from rodents housed in their home cages, but currently they are unscalable (Stiedl et al., 1999; Kramer and Kinter, 2003; Gaburro et al., 2011; Kuzdas et al., 2013).

### Science-Driven Need for Refinement and Evolution of Animal Assessment Technologies

The technologies described above that allow for the automated, unrestrained measurement of behavior and physiology continue to have an enormous impact on preclinical research. Many traditional behavioral tests have proven utility and validity, and have contributed much to today’s knowledge of the regulation of locomotion and other behavioral endpoints, such as anxiety-related behaviors. However, these traditional behavioral and physiological tests have limitations, as they occur in a dedicated testing apparatus during a short period of time [or require invasive surgical manipulation for instrumentation (i.e., telemetry)] and most often with singly housed animals.

There are animal welfare and scientific validity issues related to assessments occurring in dedicated testing apparatuses. For example, because of their limited time period, relevant dynamic and circadian processes cannot be included. Furthermore, true baseline information can be difficult to obtain because of both the novelty of entering a testing apparatus and the physiological changes that result from handling stress. These factors can obscure the behavioral phenotype one seeks to understand (Chesler et al., 2002; de Visser et al., 2006; Pernold et al., 2021). In fact, even in a home cage, handling mice for routine husbandry procedures (e.g., weighing or cage change) can increase the hormone corticosterone and daytime activity for at least 24 h (Rasmussen et al., 2011; Pernold et al., 2019). Finally, different experimenters and/or experimental conditions (e.g., environment before, during, and after experiment) can also play a pivotal role in data reproducibility (Pernold et al., 2019; Richter, 2020). Even in human and veterinary medicine, the presence of an experimenter can induce experimental confounds, i.e., “white coat effect” (Mattoo et al., 1976; Lavie et al., 1988; Stanek and Bruckner, 1989; Bodey and Michell, 1997; Pioli et al., 2018).

Single-housing of animals is also a potential issue for animal welfare and experimental validity. Single-housing alters animal behavior, limits the expression of natural social behaviors (Kappel et al., 2017; Arakawa, 2018; Manouze et al., 2019), and changes physiological parameters (Kerr et al., 1997; D’Amato et al., 2001; Van Loo et al., 2007; Hermes et al., 2009; Pham et al., 2010; Haj-Mirzaian et al., 2019). Furthermore, The Guide for the Care and Use of Laboratory Animals (The Guide) states that social animals, such as mice and rats, should be housed in stable pairs or groups of compatible individuals unless single-housing is required for scientific reasons or social incompatibility [National Research Council (US) Committee for the Update of the Guide for the Care and Use of Laboratory Animals, 2011]. Therefore, any data collected from singly housed animals should be carefully scrutinized.

Instead of taking an animal to an experiment, experimenters could instead refine testing by bringing the experiment to the home environment of the animal to minimize the confounding variables and animal welfare concerns mentioned above (Richter et al., 2010; Voelkl et al., 2018, 2020). Assessing animals in their home environment allows for long-term continuous observation, with establishment of baseline activity followed by programmed interventions (Tang et al., 2002; Kas and Van Ree, 2004). Home cage assessment requires minimal human intervention, which reduces handling stress and experimental bias. It also increases operational efficiency by reducing time required for humans to make observations (Tecott and Nestler, 2004) and for animals to become acclimated to a novel apparatus. By designing a home cage environment as an automated, modular system that contains different stimuli (e.g., food, drink, light and sound stimuli, novel objects) and enrichment (shelter, play objects), a broad range of behaviors, as a result of interacting motivational systems, can be studied (de Visser et al., 2006; Voikar and Gaburro, 2020). It allows for the distinction of novelty-induced and baseline behaviors and offers the opportunity to study circadian rhythmicity and sleep alterations.

In order to turn a home cage into an automated behavioral and physiological assessment system, a variety of sensors [e.g., video camera, RFID and electromagnetic field (EMF) sensing boards, and vibration sensors] must be added to the home cage. Digital rodent longitudinal monitoring technologies, scalable and unscalable, have been described previously by Voikar and Gaburro (2020). This manuscript complements the Voikar and Gaburro (2020) review by providing a questionnaire with suggestions of additional descriptive information to be collected from technology providers (Table 1). Collectively, this information will assist end-user with selection, onboarding and resource planning when considering DBs, data accessibility and visualization.

**TABLE 1:** Questionnaire with suggestions of descriptive information to be collected from technology providers to assist end users with selection, onboarding, and resource planning; **(A)** general overview and data accessibility and visualization, and **(B)** digital biomarkers.

			Company A	Company B	Company C
**General information**					
Technology type (EMF, RFID, Telemetry, Wearable, Video, Other)					
Number of video cameras per cage/system, if applicable					
Location of cameras, if applicable (Side, Top, Other)					
Data storage type (Local, Cloud, Hybrid)					
Type of data (Image, Numerical, Video)					
Amount of data per one system or cage per 24-h period (GB)					
Species					
Implant size (mm), if applicable					
Animals per one system or cage (specify species)					
Home cage compatible					
Rack compatible					
Rack based					
Scalability; Low (1–80 cages), Medium (81–180 cages), High (181 and more cages)					
**General data accessibility/visualization**					
Raw data accessibility					
Web browser capability (direct, without app)					
Application capability (application has to be downloaded?)					
Availability of data to the user (in minutes); *(Delay b/w viewing animals live in vivarium seeing automatically extracted biomarkers through your platform?)*					
Individual animal data when socially housed					
Group housed					
**Automated dashboard data comparison options (this excludes manual comparisons of data)**					
Individual					
Group					
Strain					
Sex					
Light cycle (Day vs. Night)					
Activity distribution					
Time of the day/week (min,h,day)					
Percent change given parameter vs. baseline					
Descriptive stats of parameters (Mean, Avg, STDEV, SEM)					
Enviromental factor (%Rh, T, Light, Humans, Vibration)					
Zooming into areas of interest on data dashboard					
Resolution options (seconds, minutes, hours) *(View data every 30 s vs. 5 min)*					
Tasks on subject charts (include manual observations)					
Data analytics (Locally based, Cloud based)					

			**Company A**	**Company B**	**Company C**

HA	Health Alert	Health alert functionality			
Physiology	Physiology	Temperature			
		Respiration			
		Blood Pressure			
		Heart Rate			
Behavioral	Consumption	Water (time spent)			
		Food (time spent)			
		Water (actual			
		Food (actual)			
	Motion	Velocity			
		Distance			
		Total Movement			
		Direction			
	Activity – General	Climbing			
		Rearing			
		Foraging			
		Self-Grooming			
		Allo-grooming			
		Scratching			
		Writhering			
		Jumping			
		Sleep			
	Activitiy - Aggression	Pinning			
		Pouncing			
		Sliding			
		Bumping			
		Dominant Grooming			
		Biting			
	Running Wheel Related Behaviors	Time on wheel			
		Velocity			
		Distance			
		Direction of wheel rotation			
		Frequency & duration of bouts			
		Consistency of wheel velocity			
		Multiple mice on wheel			
	Cage Zones	Time spend in zone			
		Speed in zone			
		Number/duration of boughts in zone			
		Transitions between zones			
	Social Trajectory Analysis	Distance between animals/social distance			
		Time spent together			
		Following behavior			
		Exploration index			
		Thigmoaxic behavior			
	Other	Convulsion			
		Seizure			
		Tremur			
		Circadian Rhythm			

### Potential 3Rs Impact of Digital Biomarkers

The internationally accepted principles of the 3Rs in preclinical research were first published in 1959 (Russell and Burch, 1959). Since then, they have become internationally accepted principles of humane and ethical science. DBs have the potential to support the reduction and refinement principles of the 3Rs.

There are several ways that DBs can support refinement. DBs can be used to assess the effectiveness of efforts to improve animal welfare by decreasing pain and distress, such as administration of postoperative analgesia (Roughan et al., 2009). They could also allow researchers to more quickly and accurately detect and track either experimentally induced or naturally occurring diseases and ailments. Presumably, they could even detect subclinical (mild) disease and cases where the animals may be distressed but not showing visible symptoms (Lim et al., 2017; Baran et al., 2020, 2021). They could even more accurately predict end of life. Therefore, this could allow for earlier intervention, reducing animal pain and discomfort, morbidity, and mortality. These biomarkers also have the potential to replace traditional measurements, such as blood collection, that require handling and resulting in pain and distress, allowing for further refinement of procedures (Steele et al., 2007). They could even be used to evaluate effective methods to promote positive welfare by promoting positive states (Yeates and Main, 2008; Mellor, 2012).

Although there are many examples of using DBs to refine animal studies, we will outline two particularly clear examples. The first example is where an automated home cage monitoring system was used to evaluate the applicability and severity of a frequently used acute colitis model (Zentrich et al., 2021). Using this system, researchers were able to closely examine the progression of colitis longitudinally in a contactless, objective, continuous, and non-invasive manner. Reduced activity, a sign of colitis severity, was observed with gold standard clinical parameters and detected with DB. The researchers concluded that such a system can be used for large-scale objective severity assessments to refine both animal welfare and scientific quality.

A second example of using DBs to refine animal studies is *via* the Cognition Wall paradigm, which is used to test discrimination and reversal learning in mice. The field of Alzheimer’s research can be complex and challenging to translate to humans while simultaneously being time-consuming and stressful to the animals. However, the Cognition Wall paradigm can be implemented to reduce time and animal stress. This paradigm can be implemented in a specially designed experimental cage in which the animals spend several weeks. This cage is equipped with a plastic insert with 3 holes, an overhead video camera, a sucrose pellet dispenser, an overhead video camera, and a software script. Mice learn that they will receive a sucrose pellet when they move through the holes in a certain pattern. Sucrose pellets are delivered automatically via the cage equipment and software script (Heldring, 2019; Grieco et al., 2021). Wild-type mice learn this very quickly: at 12 weeks of age, they reach 80% correct entries within 6 h (i.e., within a single night). At the same age, APP/PS1 mice need 50% more trials to reach the same criterion. The learning deficit can be rescued by administration of the BACE1 inhibitor LY2886721 (Heldring, 2019). This study demonstrated that with the use of specific behavioral biomarkers, Alzheimer indicators (cognitive defects) can be detected at an early stage, even before the onset of plaque pathology (Aß deposition) in the brain, and much earlier than the age at which learning impairment is detected in the Morris water maze (9–12 months). This implies a three-fold reduction in the time the mice have to be kept in the vivarium. Furthermore, the Cognition Wall test is less stressful than the Morris water swim task.

There are also several ways that DBs can support reduction (Vollert et al., 2020; Iman et al., 2021; Zentrich et al., 2021). In-person animal assessments, including manual score sheets, are an important issue, as they are subjective and introduce inter-assessor variability with potentially serious implications for reported outcomes. Objective biomarkers address this challenge and can enable stronger repeated-measure experimental designs with more sensitive detection of variation induced by treatment. Repeated measures can reduce the need for multiple satellite groups of animals that are sacrificed at various points for histopathological assessment of disease progression (Baran et al., 2020). Furthermore, these biomarkers could mitigate variability among human raters, thereby allowing greater precision. Home cage behaviors that predict disease onset (Steele et al., 2007) could also guide researchers to more physiologically relevant and robust endpoints that could inform planning of future clinical stage trials. This knowledge, in turn, allows for better experimental study designs and promotes collaboration and coordination among scientists. These qualities of method development, coordination, and planning of animal experiments have been shown to be the three main qualities that contribute to the effective application of the principles of the 3Rs (Törnqvist et al., 2014).

## Limitations and Barriers to Implementation

Despite the potential value of translational DBs to science and the 3Rs, there are clear barriers to implementation that need to be addressed. Here, we describe operational, scientific, and cultural barriers. Some of these barriers are not unique to DBs, such as fear of change; therefore, we will not discuss them extensively. However, we will discuss the ones unique to DBs, such as digital infrastructure, in greater details. Overall, early and careful consideration of these topics will help ensure successful implementation of DB technologies.

### Operational

#### Information Technology Infrastructure

Technologies that make DBs possible are composed of hardware and digital platforms.

Digital platforms can be on-premises or cloud-based as Software as a Service (SaaS). For on-premises infrastructure (private cloud) or Infrastructure as a Service (IaaS, public cloud, such as Amazon Web Services (AWS) or Google Cloud Platform (GCP), infrastructure needs are handled by an institution team. For SaaS, infrastructure needs are handled by a digital technology vendor. Each option comes with associated benefits and costs. On-premises technologies rely heavily on internal information technology (IT) skills and support. As companies drive toward lean and more efficient operational models while increasing digital engagement, competition for internal IT resources can be fierce. After obtaining this support, it can also be challenging to maintain the required level of IT support for DBs. SaaS technologies, while offering scalability and flexibility, require digital infrastructure to allow for data flow and potentially significant bandwidth between hardware components and the cloud (especially for video data).

The infrastructure should reflect expectations and business needs. That is, most would expect quick access to recently measured data. However, large datasets require network transfer and processing. To mitigate this issue, an ideal system would make some information instantly available for monitoring, while full large datasets would be available later for further analysis after processing. As some of these systems create massive datasets, and some enable near real-time alerting and intervention, or both, it is especially important to consider connectivity reliability and how systems may alert users when connectivity issues occur, such as when systems stop working, for example, if a cloud-based system being used by a company to collect study data is suddenly disconnected because a network team supporting the network security of the company ran a security certificate update, thus blocking data uploading to the cloud.

While these issues may not be fully preventable, it is necessary to consider real-time power backups, local data storage, and data storage backup plan. To reduce lost data and resources, vendors and end users need an understanding of how to detect when issues arise and have appropriate channels for communicating and addressing issues while using local data storage.

Additionally, edge computing can be beneficial, such that image processing is performed close to the sensor and only extracted metrics are sent over the network. For example, Nvidia Jetson Nano embedded inside an instrumented cage can run image processing software, or AWS Panorama Appliance can perform machine learning in the data acquisition location. The goal is to provide centralized control and decentralized execution, the same environment to develop, connect, manage, secure with the same tool from the edge to the cloud. Same services are available with reduced latency and lower cost. Multiple solutions are available depending on business needs, but the architecture needs to be part of the planning phase before deploying these digital technologies. One can have multiple sites for instruments (internal or external to the company) and leverage metro center edge locations (local data centers) to decrease latency concerns, or even run local sensors or computers to perform advanced analysis on premises without transferring the entire data over the network.

#### Cybersecurity

Independently of motives (intellectual property, client and patient data, extremists), life science organizations are targeted by cyberattacks (Terry, 2018; Rajagopal, 2019). It is vital for institutions to secure their data while making them available. Scientists must be able to access and share information with their peers and collaborators. However, preventing unauthorized access to data and systems while enabling science is a major challenge for organizations. Leveraging cloud technology to move quickly increases the need for cybersecurity awareness and dedicated efforts. A large amount of sensitive data is stored in the cloud and third-party-hosted environments.

Multiple strategies have to be implemented to counteract cybersecurity risks. It starts with financial and strategic investments from the leadership with a focused role, such as a chief information security officer position. The IT and security strategy needs to include roadmap items, such as data encryption, identity and access management, as well as risk and compliance (Desai et al., 2019). Standards are available in the industry to guide these strategies (National Institute of Standards and Technology, 2019), but each organization needs to adapt to its needs.

Risk framework and mitigation are critical for the infrastructure and data owners, and for working with third parties, including IT providers, data collection systems, external contractors. Reliance on systems and data, which is not under one’s control, makes it potentially more susceptible to a cyber event. Most organizations work with SaaS systems, cloud providers, software vendors, and external collaborators. It is the responsibility of the data owner to ensure that security standards and processes are in place to mitigate risks. Data integrity is critical for research, and all parties must validate the data. It is especially true when relying on additional network entry points, such as Internet of Things (IoT) devices, sensors, or wearable. These products require a network access that must be constantly monitored, making it difficult for IT to keep up with demand. Organizations are forced to choose between spreading sensitive data to third parties, evaluate the risks or lose velocity to implement scientific solutions.

Life science institutions cannot rely exclusively on technical solutions to address cybersecurity challenges. Developing a clear plan and risk mitigation strategy ahead of time can dramatically reduce the cost of an event when it occurs. It is essential to ensure appropriate resources to make sure that systems are secure and supported, and that data are accessible. All systems should be vetted through an organization’s business technology solutions group to ensure they match institutional cybersecurity and compliance policies as well as industry standards. It is important to embed operational resiliency into everyday activity: implement strong authentication to sensitive information and manage staff and third-party access, deploy specific security capabilities for secure production IT, ensure data integrity, and provide personal trusted identity to researchers with private keys (PKI) solutions.

#### Data Integration

Data integration, including and beyond integration (and synchronization) of behavioral and physiological data, is another challenge. Most companies already have a procurement and BioRegistry system in place to assign unique identifiers to animals and associated metadata. These data must be mapped to new translational DB data for data integrity and sample tracking. For example, integrating behavioral data captured in digital cages with physiological data measured at a different time requires additional engineering work.

#### Data Quantity and Flow

As discussed, some of these emerging technologies can generate a great deal of data depending on their architecture, signal processing, and data reduction workflows. This requires development of data storage strategy and retention policies. Data storage strategy should consider data format, metadata, and access frequency and timing. The flow of data should be automated, requiring minimal manual input to reduce data entry errors. Long-term raw video data storage can require significant financial support that often deters organizations from keeping this type of data. However, in the spirit of the 3Rs principles, it should be considered, since historical raw video data can be used to develop and validate algorithms to extract novel DBs.

#### Analysis and Interpretation

Analysis of continuous 24/7 data for multiple cages and multiple days is challenging, and different approaches have been proposed. Temporal data can be aggregated into packets (5 min, 1 h, etc.) linked to circadian time, and the same data (such as behavior counts) may also be filtered depending on other factors, such as the injection time and pharmacokinetics/pharmacodynamics (PK/PD) profile of a specific candidate drug, or whether the biomarker to be evaluated is evident mostly during the active phase (nighttime) of animals. Pilot studies are useful to optimize and define settings for comparison. Once these are defined, baseline activity recordings allow for the parameters of a subject to serve as its own control. Data may also be normalized in the case of uneven animal number per cage; however, this poses additional complications, particularly when trios, pairs, and singly housed rodent environments result in different opportunities for behavior, hierarchies, and social effects. This may be addressed by normalizing data against a 1-to-2-week baseline in which a parameter can be expressed as % change (increase or decrease of the activity).

Behavioral and physiological biomarkers can also be used in experimental planning to balance groups of animals based on expected behaviors. For example, baseline data could demonstrate that some subjects are hyperactive or hypoactive, which can be considered in randomization or allow for the removal of those individuals from consideration in an experiment if activity measures are already between 1 or 2 standard deviations (SDs) outside of average activity.

Descriptive statistics for continuous time-associated biomarkers are complex, and data obtained from these systems may be incompatible with classic parametric *t*-tests or analyses of variance. Consulting with an experienced statistician prior to experimental onset is recommended. In many cases, non-parametric tools are able to handle non-continuous, complex data better (Voikar and Gaburro, 2020). Interpretation of results can also be challenging, when animals are socially housed, because of signal crossover, loss of signal, or inability of a system to collect continuous data from individual animals.

### Operational and Scientific

#### Non-information Technology Resource Requirements

As different types of technologies and systems exist, scalability, operational footprint, and resource requirements (i.e., compatibility with standard cage and rack designs and additional space requirements) are key points to discuss with internal teams early in the onboarding process. Other considerations should include staff training to operate and use new systems plus the amount of time that is required to set up and incorporate new systems while maintaining current systems. Compatibility with standard cleaning and decontamination process and capabilities must be considered, since these technologies include electronics. Resource requirements for reusable vs. recyclable caging could also be considered. If cage enrichments are used as a part of a DB recording (i.e., running wheels), the operational time and cost of using such enrichments should be included in the analysis of resource requirements.

#### Data Science

**FIGURE 2:**
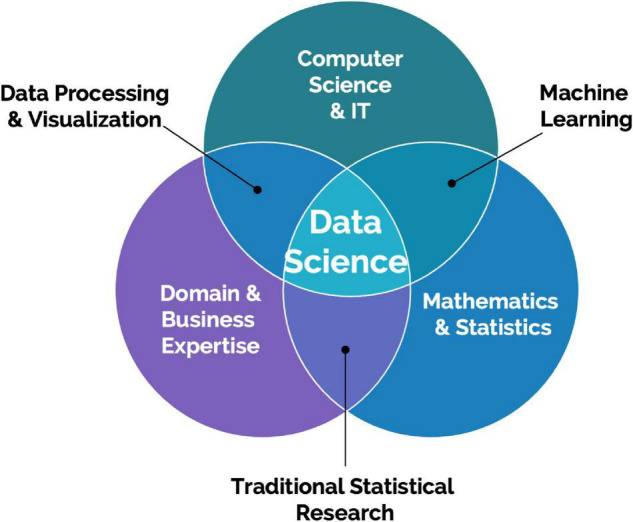
Data Science is an interdisciplinary field focused on extracting knowledge from data. It requires a combination of skills, mainly statistics and mathematics, information technology understanding, and domain knowledge.

Creating valuable insights and increasing scientific and operational value from big data collected from scalable DB technologies require data science specialization with computer vision, data processing, and advanced statistical modeling including deep learning expertise (Figure 2). These types of resources are typically not dedicated to projects involving these emerging technologies. An additional challenge is access to data scientists with both preclinical research knowledge and expertise in computer vision. Data scientists with this expertise will be able to turn data more effectively from these advanced systems into actionable insights for preclinical project teams and successfully drive collaborations with external systems and data providers. It is sometimes expected that data scientists work on collected data after an experiment is completed. However, data scientists should be involved from the beginning to work on the design of the experiments, and build the data architecture and required analysis pipeline to validate a scientific hypothesis. This prerequisite adds to the difficulty of enrolling dedicated data scientists to such projects.

#### Time From the Decision to Engage to Running Studies

**FIGURE 3:**
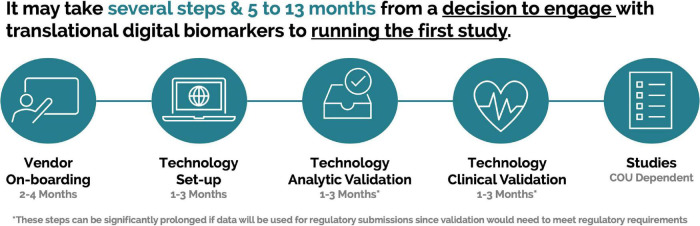
Example timeline for a pharmaceutical company from decision to engage with a scalable digital biomarker technology to running a first study.

This process within the pharmaceutical industry can take several months (Figure 3) and can be further prolonged if an organization is planning on utilizing data as part of regulatory submissions. It is important to share these timelines with all stakeholders, so that definitive projections can be developed to allow for accurate support and delivery.

#### Technology Verification and Validation

Technology verification ensures that a device is measuring and storing data accurately. This process includes demonstration of precision, reliability, and reproducibility, and, most often, is completed by a technology manufacturer. Validation is composed of analytical and clinical components (Goldsack et al., 2020).

Analytical validation entails the evaluation of data processing algorithms that convert technology-collected measurements into outputted metrics. This part of validation is also commonly performed by technology providers. Until recently, assurance from technology providers regarding verification and validation were taken at face value. However, as the technology is maturing, end users are requesting verification and validation data and insight into algorithms. Some institutions are even developing technology agnostic validation platforms (Syllable Use Cases, 2021). These aspects can be challenging, because verification process and analytical validation portions are often proprietary.

Clinical validation is accomplished by demonstrating that technology adequately identifies, measures, or predicts a meaningful clinical, biological, physical, functional state or experience in both the specified animal cohort and context of use (Goldsack et al., 2020). When gold standard measurement is available, head-to-head comparison should be conducted to determine sensitivity and specificity. This validation is time-consuming, and during these early stages of technology, maturation often needs to be performed by end users. This challenge provides another justification for an internal data scientist who could successfully drive collaborations with external systems and data providers, some of which provide poorly validated black box algorithms. Robust assessment of reliability, reproducibility, and usability (user experience) should be performed.

### Scientific

#### Consideration and/or Understanding of Technology Impact

##### Cage Enhancements

In these early stages, the full impact of these technologies on models is not well understood. In the same light, if cage enhancements (i.e., running wheel) are performed, the impact on model development and study design should be balanced with potential outcomes. For example, running wheel activity as a critical marker of quality of life versus the impact of exercise on metabolism, immune function, behavior, and the standard model (de Visser et al., 2005; Devisser et al., 2007). While change in model outcomes may be considered a barrier to implementation, it could also lead to a more biologically relevant or predictive model. It is crucial to be aware of these potential confounding factors. Current published literature on running wheel behavior is inconclusive, and there seems to be a varying degree to which voluntary exercise affects home cage behavior that might impact disease models (Sherwin, 1998; de Visser et al., 2005; Devisser et al., 2007). This raises an interesting question about introducing inherent variability into our preclinical models. Perhaps a model that provides half of the animal’s access to exercise provides a more clinically relevant model, considering that not all human patients have the same activity levels, housing, or diet (Richter et al., 2010). It can be challenging to accurately translate the use of the running wheel and what that biomarker reflects in addition to wheel activity. However, as a single traditional measure is not sufficient on its own to interpret, same is applicable to DBs.

##### Cage Requirements

Other considerations include how standard housing and optimal welfare may be challenged or changed by system requirements. For instance, some systems may require single housing or modified enrichment (decreased or no nesting material) to enable recording of animal behavior and/or obtaining individual animal data. So not only does this require ethical consideration of the impact on animal welfare but also of the potential impact on study outcomes.

##### Health Alarms

Since selected technologies have the ability to send out health alarms, it is necessary to consider how this information will be interpreted and potentially acted on, for example, if a veterinary technician comes to a site and intervenes if a health alarm is received in the middle of the night.

#### Understanding Measurements and Associated Metadata

As with any assessment, it should be considered and understood what is being measured, for example, when a scientist requests to assess the sleep cycle, activity, and/or motion of an animal, these cover a broad range of possible measures. Sleep cycle assessment could include time to fall asleep, time to wake up, total sleep time, time in and outside of nest, and group and individual sleep measurements. Activity assessment could include local or moving exploration, eating, drinking, standing, rearing, or grooming, and motion could include velocity, distance, total movement, direction, stride characteristics (count, duration, and cadence), and gait characteristics. Understanding what assessment is being requested and knowing what is actually being measured will assist with accurate data interpretation and evolution of technology.

#### One Size Fits All

When looking for solutions, such as digital solutions, initial inclination is to identify a solution that can be broadly used. This leads to high expectations and pilots designed with broad applicability. This approach to these emerging technologies is challenging, as they should be piloted with a COU design during their early development and engagement. Since each technology and measurement offers can be very different (Tables 1–3), technology comparison and selection, without COU in mind, can lead to engagement with an incorrect system.

**TABLE 2:** Barriers and solutions to implementation of scalable digital biomarker technologies that end users can implement.

Barrier to implementation	Possible Solutions
Information Technology (IT) and infrastructure to support technologies.	Engage with IT, obtain infrastructure white papers or similar documentation from the technology provider and perform gap analysis of internal infrastructure early in the process.
Cybersecurity	Engage with cybersecurity team and obtain data flow white papers or similar documentation from the technology provider as early as possible.
Non-IT resource requirements	Involve both vivarium operations leadership and scientists in the selection and planning of new technology, and vivarium staff in the implementation of new systems. Invest in training in the operation of such systems.
Communication between technology provider and end-user	Identify main point of contact for technology provider and for end-user. Prior to onboarding develop a project plan, including deliverables and timelines.
Consideration and/or understanding of technology impact on *in vivo* model or biology	Collate relevant publications demonstrating the benefits of technologies and present to the team. Identify publications with specific performance data (heat load, near-IR radiation, ultrasound) to inform decision making.
Consideration and/or understanding of changes in animal housing on *in vivo* model or biology	Identify publications and performance data addressing impact of single housing, presence of running wheel, decreased or absence of nesting material. Assess effects through pilot studies, build variability into study design. Meet with colleagues or other end-users to discuss costs, benefits, and impacts to their model.
Social housing and data gaps	Learn if there is loss of data when animals are group housed and if individual animal data is available when animals are group housed. Engage data scientist to assist with data analysis. Engage with technology provider to identify most appropriate per cage animal density.
Data quantity (e.g., one hour of High Definition video is ~39 Gigabytes (frame and codec depending), on a rack with 50 cages it is 1.95 Terabytes	Map out data flow and develop data storage infrastructure, maintenance, access strategy including data retention policy. Identify capability to visualize, including ability to making comparisons across large and complex sets of data.
Time from the decision to engage to running studies	Map out realistic timeline and share with all stakeholders.
Technology verification and validation	Establish guidelines for how novel digital biomarker technology should be validated; as an example, methodologies to compare digital measures to more traditional measures can ease the uptake of emerging technologies by scientists.
Study design	Involve dedicated data scientists upfront to improve study design taking into account n number (cage or animals, depending on the technology) and relative power calculation for the outputs to be expected.
Regulatory application of a novel biomarker	Engage health authorities early to identify COU and qualification criteria and co-develop publications.
Fear of change	Educating teams about digital monitoring technologies, 3Rs benefits, and study approaches with an understanding that some approaches might fail.
Collaboration	Internally, establish mechanism or group to aggregate experiences of studies with these technologies such as Knowledge Exchanges. Industry wide, establish precompetitive groups with various stakeholders such as the Translational Digital Biomarkers Initiative within NA3RsC to share their experiences and serve as a knowledge repository with a goal of establishing more universal approaches to these emerging technologies.

**TABLE 3:** Suggested list of pragmatic information end users should consider prior to onboarding of scalable monitoring digital technologies.

- Initial set up timeline - Budgets and timing for initial trials to acceptance - Capacity - Scalability - Maintenance required - Space requirements - Equipment requirements - Digital platform requirements - Level of training and expertise required for hardware and digital platform - Cleaning and decontamination feasibility - Technology providers willingness to engage in trials and future capability build out - Institutional commitment - Degree of applicability and value across the enterprise - Use of comparable measures in clinical setting - Depreciation considerations	- Data ° Collection ° Storage ° Management ° Integration ° Curation ° Visualization ° Access ° Data analysis including ability to apply machine learning and AI ■ Individual ■ Multiple ° Historical - Model (algorithm) ° Development ° Deployment ° Maintenance	- End-user ° Mobility ° Ease of use - Facility Infrastructure ° Access, location and number of ■ power outlets ■ internet ports - Access Infrastructure ° Network speed ■ Hardwired ■ Wi-Fi ° Network access ° Location ° Hardwired vs. Wi-Fi - Hardware access ° In facility ° Outside of facility - Hosting ° On-premises ° Cloud ■ Private ■ Public ■ Hybrid
		

#### Study Design

The way our models are set up and how we run our studies need to be carefully designed with these systems. See Practical Strategies for Implementation for study design considerations.

### Cultural

#### Fear of Change

Fear of taking risk, technology failure, and stepping outside of traditional and well-established processes are common barriers to implementing new technologies.

#### Collaboration

Initial engagement with emerging technologies often happens within one group that later learns that other groups are interested or are working with similar technology. Working in functional and departmental silos leads to duplicative efforts. It makes it challenging to form feedback that makes it difficult for technology providers to act on a single view of end users.

As scalable DB technologies evolve and their adaption increases, the implementation pathway of this new approach to data collection will become more defined and robust. Since we are not there yet, here, we describe practical examples of impact and strategies for adaptation and considerations how end users can implement these emerging technologies.

## Addressing Limitations and Barriers, Practical Strategies for Implementation

There are a variety of ways to mitigate the challenges discussed above. However, we recommend particular questions and approaches at each step in the implementation process. There are also several published studies that can be used as guides to enable faster validation and qualification of studies using DBs. Overall, any study conducted must follow standard scientific practices while including additional considerations to ensure high-quality DB data.

### Step 1

For highest impact, identify studies in which traditional study measures do not meet scientific or welfare needs. DBs have been explored as a method to overcome a variety of challenges with traditional study measures. The following are examples of current problems that could be addressed using translational DBs.

• Measures that are not predictive or reproducible between studies/labs, and do not translate to clinical outcomes.
• Measures that require extensive training or large number of animals per group for statistical significance, or require animals to be in significant pain/distress to measure significant differences between groups.
• Longitudinal disease tracking, especially in diseases with variable onset and rates of disease progression.
• Models with unexpected mortality (e.g., health that declines rapidly without obvious warning signs or so slowly changes is difficult to assess).
• Measures that are sensitive to the emotional state of animals and change with disturbance.
• Situations where modest therapeutic improvement would be clinically relevant, but current measures are not sensitive enough to detect complete rescue.
• Models where baseline animal behavior/physiology can impact disease induction or variability.

### Step 2

Run a pilot study using a traditional design to collect DBs alongside traditional measures while making minor modifications to account for potential confounding effects of husbandry and study procedures. Regardless of whether an animal model is familiar or new, plan to run an initial pilot study using a traditional study design. A traditional design must include a control group and a disease group, traditional measurements (e.g., body weights, joint measurements, histology, etc.), and be properly powered to enable statistical significance testing for traditional measurements. By running a traditional study first, scientists are able to both confirm expected study outcomes and begin to explore the ability of DBs to solve pre-identified scientific needs.

As outlined before, routine husbandry (e.g., cage change) and study procedures (e.g., blood draws) can have a significant impact on animals, and can last for multiple days (Rasmussen et al., 2011; Lim et al., 2019; Pernold et al., 2019; Baran et al., 2021). In designing a “traditional” study, it is important to modify the timing of procedures to consider these potential confounding effects. Identify all procedures that will involve human-animal interactions and identify a time to conduct the procedure that will not overlap with key data collection time points. For example, in the cuprizone study described in Lim et al. (2019), it would have been prudent to schedule cage change 4 days earlier to avoid washing out of the signal associated with remyelination. Given the potential confounding effects of procedures, be sure that all animals are exposed to the same study procedures at the same time.

Finally, if a study design calls for induction of disease on healthy animals (e.g., paraquat lung injury, peripheral neuropathy, etc.), plan to collect > 6 days of TDB data to use as a baseline for randomization the day prior to disease induction. It is recommended that scientists perform stratified randomization, a strategy that allows researchers to control and balance animals in groups based on key study measures, in this case TDB.

### Step 3

Analyze pilot study data to uncover the potential value of TDB. A number of analysis strategies can be used to determine the added value of TDB. The following questions can help guide exploration. Do TDBs enable new insights into a disease model? For example, in a paraquat model of lung injury, digitally collected breathing rates enabled tracking of disease progression and improvement with therapeutic bardoxolone (Baran et al., 2020). Do DBs track with expected disease progression? If the goal is to eventually replace a traditional study measure, looking at correlations between traditional measures and TDB may be helpful. For example, in ALS mice, digitally collected rest disturbance indexes are highly correlated with grid hanging and body weight (Golini et al., 2020). Are DBs more consistent than traditional measures? Some study measures that rely on manual collection by an experimenter are more susceptible to inter-experimenter variability, such as joint thickness in arthritic animals or tumor volume in oncology studies. Lim et al. (2017) demonstrated that a DB can be created that more accurately measures disease in a rat model of rheumatoid arthritis than standard joint measurements. Be sure to take note of behavioral responses immediately surrounding husbandry and study procedures for confounding effects on data interpretation as well as potential insight into phenotypes. Often, TDB can provide an additional layer of information that can help better interpret more traditional study measures. During this review, you may uncover unexpected challenges with your pilot study design. If necessary, plan to run a follow-up study.

### Step 4

If pilot study data suggest TDB may be of value, run a follow-up complete study to verify reproducibility. At this point, preliminary data may suggest that TDB can provide you with complementary or, perhaps, more meaningful data. To be valuable, it will be necessary to show that these data can be reproducibly obtained from multiple studies. Conduct a follow-up study using the exact same study design (including procedure schedule), but in addition to the control and disease groups, it is ideal to include one or more therapeutics with known efficacy. This design allows scientists to both validate the ability to reproduce TDB results and assess the ability of a known therapy to move a TDB. Note that to ensure reproducibility, be sure to use the same statistical strategy to analyze both studies.

### Step 5 (Optional)

If collaborations are anticipated, ask a collaborator to replicate your findings. The ability to replicate findings across highly variable laboratory settings will further serve to confirm the reproducibility of TDB.

## Addressing Limitations and Barriers

**FIGURE 4:**
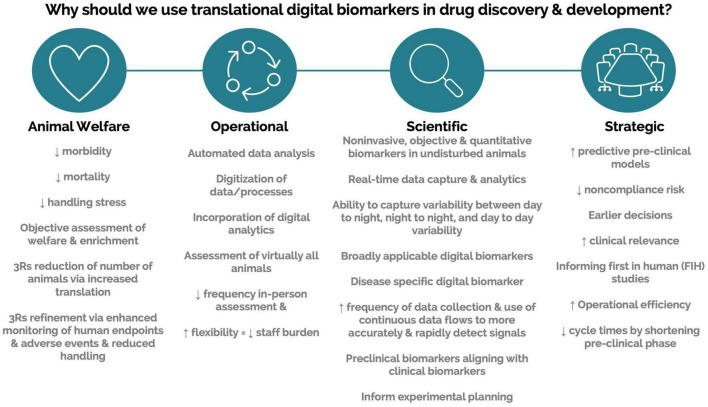
Value propositions for translational digital biomarkers within drug discovery and development.

Engagement with scalable DB technologies requires interaction across multiple areas of expertise. Driving engagement and obtaining support require the ability to communicate potential value across a variety of stakeholders. Here, we compiled a list of value propositions that can be used when presenting these technologies to various stakeholders (Figure 4). We also list barriers to implementation and possible solutions (Table 2). Finally, we compile a list of topics and questions that should be considered when onboarding these emerging technologies (Table 3).

## Conclusion

The digital age has paved the way to automate and objectively measure animal behavior and physiology. As more knowledge is gained of biological systems, there are more possibilities to digitalize aspects of animal health, function, and physiology, and explore therapy guidance and disease progression. Recent advances in scalable DB technologies have the potential to improve assessment of safety and efficacy by reducing variability while also increasing precision and sensitivity. This is because these assessments are objective (not impacted by perceptual biases), have high resolution (collected continuously), and realistic (collected within the home environment of animals).

**FIGURE 5:**
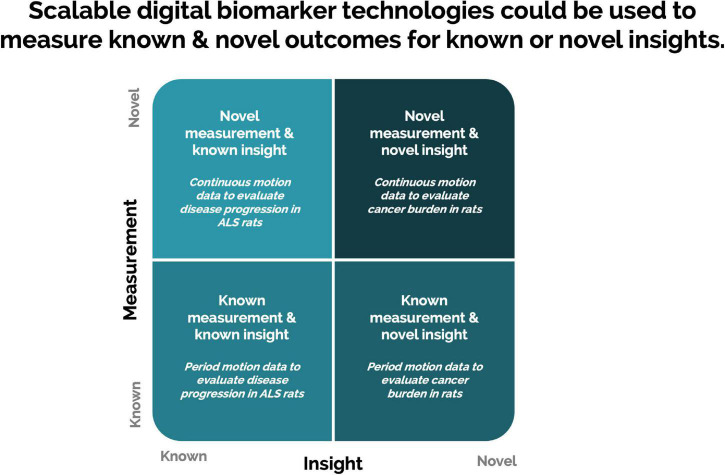
Scalable digital biomarker technologies present an opportunity to digitize the collection of traditional biomarkers and measure novel digital biomarkers (Wang et al., 2016).

**FIGURE 6:**
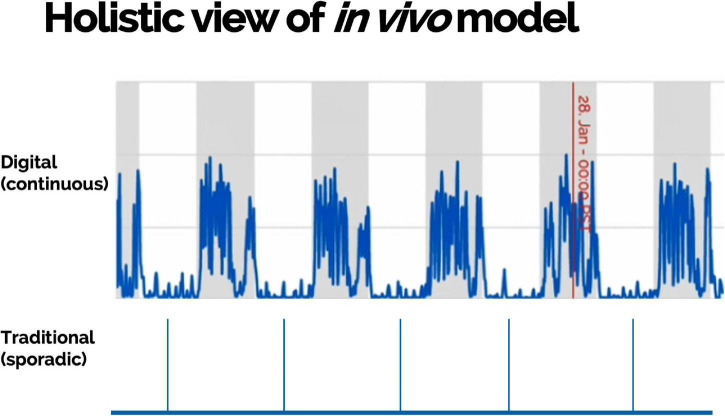
Multiple measurements collected continuously and remotely within animals’ home environment can provide a holistic view of an *in vivo* model including objective assessment of disease development and burden.

Scalable DB technologies present an opportunity to capture meaningful, objective data leading to actionable insight into animal welfare, animal tracking, study design optimization, and control on sources of variations (Figure 4). These technologies present an opportunity to measure novel DBs and digitize existing biomarkers (Figure 5). Furthermore, continuous monitoring of animals within their home environment enables a holistic view of an animal instead of the snapshot received from traditional data (Figure 6). Furthermore, it allows for the measurement of spontaneous behavior and the ability to detect subtleties in behavior that often go unnoticed by gross cage side observations (Figure 5).

In most cases, DBs also contribute to the promotion of the 3Rs, such as, refinement and reduction, by serving as markers of animal welfare, providing precision, and instilling additional meaning to *in vivo* research. More opportunities exist for refinement of methodologies and application of DBs, and the advent of artificial intelligence and machine learning has led to measurements of many of these parameters in an operator-independent manner. When used correctly, a well-validated biomarker can be utilized to guide research on disease progression, as well as drug efficacy, safety, and toxicity.

Pertinent and timely data are required for a translational biomarker to inform clinical and preclinical researchers in a usable manner. To achieve and standardize such a translational biomarker requires a great deal of time and resources in order to establish its credibility as a measurement. There are a variety of devices that currently address different aspects of animal welfare, all measuring varying sets of DBs, and these are used to provide data to inform scientists, managers, and technicians of various aspects of *in vivo* health, welfare, disease status, and treatment efficacy. In addition, there are rigorous standards in place that govern data storage and protection, which must be taken into account when designing an architecture that promotes the ability to store, retrieve, and interact with large amounts of data. When onboarding these technologies, it is important to use a vetting process that includes pilot studies and data management plans.

In an effort to enhance the information obtained from *in vivo* studies and place it within a larger context of a complete drug development program, the impact of DBs cannot be understated for their compatibility with artificial intelligence and machine learning approaches to drug discovery. Creation of digital data sets that are temporal in nature can allow for acute insight into how animals act in the longer term, under specific circumstances and test articles. Digital data sets also lend themselves well to AI approaches that combine data from all aspects of preclinical and discovery stages with clinically derived data sets to contribute to AI modeling of disease in order to aid drug discovery without the use of animals (Zhu, 2020). While machine learning and advanced analytics mining large data sets are shaping the future of research, emerging technologies, such as scalable DB systems, will transform research and discovery.

Currently, we are faced with cultural, operational, and scientific challenges associated with implementation of these technologies. For these emerging technologies to be broadly implemented, they need to demonstrate additional value to science and business when compared to traditional assessments of animals. We believe that our recommended solutions will enhance technology engagement through appropriate planning prior to onboarding, technology evaluation, and implementation. Introducing a novel technology into traditional business operations necessitates top-level executive support. The value proposition, barriers, and considerations communicated here should assist with engaging leadership to provide such support.

Within drug discovery and development, the ultimate goal of preclinical research is to model a human disease state in order to better predict potential drug toxicities and treatment efficacy in the clinic. While the technologies discussed here for monitoring preclinical DBs are still in the early stages of development and implementation, this review is designed to improve the understanding of the value of DBs technologies while exploring strategies to speed their implementation within preclinical research, so that as a scientific community we can more rapidly get better therapeutics to patients in need.

## Author Contributions

SB developed the initial manuscript outline and drafted sections “Introduction,” “Potential 3Rs impact of digital biomarkers,” “Limitations and barriers to implementation,” and “Conclusions” as well as Figures 5, 6. NB contributed to the outline of the initial manuscript, drafted section “Addressing limitations and barriers; Practical strategies for implementation,” and provided review comments on sections “Introduction” and “Potential 3Rs impact of digital biomarkers”. JD reviewed the final manuscript by contributing ideas, feedback, and references. EK contributed to the first draft of section “Addressing limitations and barriers; Practical strategies for implementation” and revised the Figures and Tables 2, 3. SG and GR contributed to writing the section on digital biomarkers, and contributed to compiling Figure 1 as well as Tables 1, 2. SM revised the initial draft. LN wrote the first draft of section “Evolution of digital biomarkers, from short-lasting tests to longitudinal assessment in the home cage” and designed Figure 1. YP contributed to section “Operational & Scientific”. MR contributed to the Figures and Tables, and editing. LS wrote the first draft of section “Addressing limitations and barriers; Practical strategies for implementation”. PS wrote a first draft of the conclusion and section “Potential 3Rs impact of digital biomarkers” of the manuscript. ML contributed to directing the project and revised all the Figures and text. All the authors contributed to the final version of the manuscript.

## Author Disclaimer

This article reflects the views of a co- author JD and the other authors, and it should not be construed to represent the views or policies of the FDA.

## Conflict of Interest

SB is employed by Novartis Pharmaceuticals Corporation. NB and PM are employed by AbbVie Inc. NB and PM also own AbbVie stock. SG and GR are employed by Tecniplast S.p.A. EK is employed by Calico Life Sciences LLC. SM is employed by GlaxoSmithKline USA. LN is employed by Noldus Information Technology BV. YP is employed by Tessera Therapeutics Inc. MR is employed by Unified Information Devices Inc. LS is employed by Recursion Pharmaceuticals Inc. PS is employed by Actual Analytics Ltd and Naason Science Inc. The remaining authors declare that the research was conducted in the absence of any commercial or financial relationships that could be construed as a potential conflict of interest.

## Publisher’s Note

All claims expressed in this article are solely those of the authors and do not necessarily represent those of their affiliated organizations, or those of the publisher, the editors and the reviewers. Any product that may be evaluated in this article, or claim that may be made by its manufacturer, is not guaranteed or endorsed by the publisher.

## Footnotes

1
www.na3rsc.org
